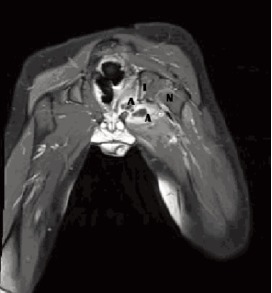# Obturator Internus Pyomyositis in A Child: A Case Report

**DOI:** 10.5704/MOJ.1403.004

**Published:** 2014-03

**Authors:** XL Chong, M Ashik, M Arjandas

**Affiliations:** Department of Orthopaedic Surgery, KK Women's and Children's Hospital, Singapore; Department of Orthopaedic Surgery, KK Women's and Children's Hospital, Singapore; Department of Orthopaedic Surgery, KK Women's and Children's Hospital, Singapore

## Abstract

Obturator internus pyomyositis is a rare disease that is more commonly found in tropical countries. Due to its infrequent occurrence, it is a differential of hip pain that has not been sufficiently considered, which often results in delayed diagnosis. We present a case report of a 4 year old boy with pyomyositis of obturator internus as well as externus. He was treated successfully with intravenous antibiotics. A comparison is made with other case reviews to identify symptoms and signs that could help in diagnosing the condition early and accurately so as to initiate intravenous antibiotics, the mainstay treatment in a timely fashion, eventually avoiding surgical drainage of the sequelae when it becomes an abscess.

**Key Words:**

Obturator internus pyomyositis, septic arthritis

## Introduction

Obturator internus pyomyositis (OIP) is a rare muscle infection. It manifests as hip pain and fever, similar to septic arthritis, a more sinister condition. When OIP is diagnosed early, it can be treated well with just intravenous antibiotics alone. Therefore, we should approach those patients with heightened suspicion as prompt diagnosis and initiation of treatment can avoid the risks of surgical drainage if OIP develops into an abscess.

## CASE REPORT

A 4 year old non-local boy was first seen by a general practitioner for a 3 day history of left hip pain and fever. There was no trauma or coryzal symptoms. On examination, he had a left hip flexion 95°, external rotation 50° and internal rotation 10°. Blood tests done on that day showed a white blood cell count of 13.2. C-reactive protein (CRP) was raised at 71.3mg/L.

He was subsequently admitted to KK Children’s Hospital Orthopaedic Department. Plain radiographs of the left hip did not show any abnormality as seen in Figure 1a and Figure 1b. Ultrasound of the left hip did not show any effusion as well.

He was started on an intravenous antibiotic, Cloxacillin, a semi-synthetic penicillin, on the second day of admission after MRI of the left hip showed rim-enhancing fluid collections involving the left obturator internus and externus muscles in keeping with abscesses, as shown in Figure 2. Subsequent blood tests showed a white cell count of 11.0 and increasing CRP of 114mg/dL. Erythrocyte sedimentation rate (ESR) was 73 mm/50mins.

He was also reviewed by the infectious disease specialist and planned to have his abscess drained if he remained febrile. On the eighth day of admission, his fever resolved and his left hip pain improved. The white cell count remained normal and CRP dropped to 57.5mg/L. He was discharged on the 9th day of admission with oral cephalexin for 5 weeks. His first outpatient review 16 days after discharge showed a normal CRP and a further drop of ESR to 43mm/50mins. His left hip remained asymptomatic and there was full range of motion. After completion of 5 weeks of cephalexin, he remained asymptomatic and was able to return to normal activities.

## Discussion

A detailed history from the patient would often yield symptoms of fever and hip pain associated with a limp. Septic arthritis needs to be excluded first, which, if left untreated could have devastating consequences. After exclusion of septic arthritis, it is pertinent to approach the patient with high index of suspicion to consider OIP as a possible differential diagnosis among other more common differential diagnoses such as transient synovitis, osteomyelitis and even malignancy.

OIP often does not limit global range of motion unlike in septic arthritis. It often restricts in a certain plane of motion. From aspects of anatomy, obturator internus and externus are short rotators of the hip. Obturator internus externally rotates and abducts the hip when it is in a flexed position. Obturator externus externally rotates the hip and stabilizes the joint. In Styles et al reported five of the 8 patients studied had painful motion but no limitation in range^1^. One patient with restricted range of motion had limited range of adduction and internal rotation but was able to flex his hip from 0 to 100°. Similarly, our patient had restricted internal and external rotation but flexion range was fairly normal. Therefore, global versus single plane restriction of motion is a good distinguishing factor between septic arthritis and obturator internus pyomyositis.

Normal CRP and ESR are good markers to rule out OIP. On the other hand, elevations of both CRP and ESR are a good indication of pyomyositis. Browne et al , found that if CRP was less than 3.6 mg/dL or ESR was more than 22 mm/hr, the patient was unlikely to have pyomyositis^2^. Raised total white count has not been found to be a good marker of OIP. Recent studies have also proven white cell count is unreliable as a marker of septic arthritis as well. Our patient did not have leukocytosis but both CRP and ESR were elevated, which favours the diagnosis of OIP.

As blood tests are not specific enough, early imaging is essential for accurate diagnosis. Plain radiograph are usually normal. Although ultrasound is a useful first line test to first rule out the more serious diagnosis of septic arthritis, MRI has been shown to be the most sensitive test in current studies.

Prompt accurate diagnosis followed by early initiation of intravenous antibiotics can prevent abscess formation and eventually avoid surgical drainage. Significant risks could include decreased range of motion due to scarring and potential inadequate drainage because of lack of familiarity with anatomy and surgical approach as well as the relatively small size of the abscess within bulky musculature. Intravenous antibiotics are the mainstay of treatment for pyomyositis if there is no drainable collection of purulent fluid seen on MRI. It should be effective against Staphylococcus aureuss, the main causative organism found in more than 50% of the bacteriology results by Wong et al^3^ It is followed by Strepoccocus pyogens and Nesseria gonorrhoea. Antibiotics should cover all three organisms.

The recommended duration of antibiotics is 2 to 6 weeks as suggested by Gubbay et al^4^. Our patient had one week of intravenous antibiotics after which his hip pain resolved and oral antibiotic was prescribed for 5 weeks. CRP returned to normal level by the end of the antibiotic therapy.

Drainage of abscess is indicated in febrile patients with pain for more than 5 to 7 days despite antibiotics or abscess evident on imaging^5^. Our patient had fever and pain, which resolved in one week’s time. Even though, MRI done for our patient suggested abscess of the obturator internus and externus, drainage was held off in view of patient’s condition stabilizing on intravenous antibiotics. After one week of intravenous antibiotics, delay in initiating surgical drainage spared the patient from operative risks as he was recovering with antibiotics alone. Therefore, despite recommended duration of persistent pain and fever as well as MRI findings of abscess as indications for surgical drainage, it is useful to assess the patient in an overall manner before making the final decision of surgical drainage.

**Fig. 1a: Plain AP radiograph of the hips.**
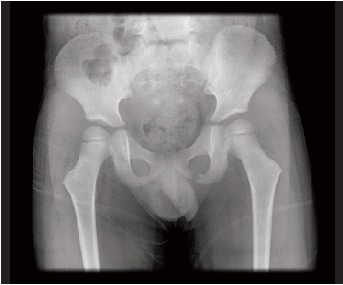


**Fig. 1b: Plain frogview radiograph of the hips.**
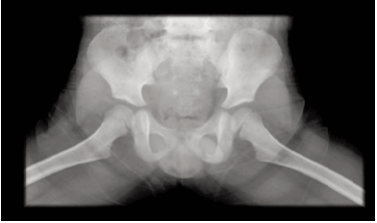


**Fig. 2: MRI of both hips with contrast T1 coronal image with contrast -, showing abscesses (rim enhancing lesions (A) arising from the inner aspect of left ischium and crossing the ischium (I). N= neck of femur.**